# Increased facial asymmetry in focal epilepsies associated with unilateral lesions

**DOI:** 10.1093/braincomms/fcab068

**Published:** 2021-04-19

**Authors:** Simona Balestrini, Seymour M Lopez, Krishna Chinthapalli, Narek Sargsyan, Rita Demurtas, Sjoerd Vos, Andre Altmann, Michael Suttie, Peter Hammond, Sanjay M Sisodiya

**Affiliations:** 1 Department of Clinical and Experimental Epilepsy, UCL Queen Square Institute of Neurology, London; 2 Chalfont Centre for Epilepsy, Gerrards Cross, UK; 3 Department of Medical Physics, Centre for Medical Image Computing, UCL, London, UK; 4 Neuroradiological Academic Unit, UCL Queen Square Institute of Neurology, University College London, London, UK; 5 Nuffield Department of Women's & Reproductive Health, University of Oxford, Oxford, UK; 6 Big Data Institute, Old Road Campus, University of Oxford, Oxford, UK

**Keywords:** brain asymmetry, dense surface modelling, facial asymmetry, focal epilepsy, networks

## Abstract

The epilepsies are now conceptualized as network disruptions: focal epilepsies are considered to have network alterations in the hemisphere of seizure onset, whilst generalized epilepsies are considered to have bi-hemispheric network changes. Increasingly, many epilepsies are also considered to be neurodevelopmental disorders, with early changes in the brain underpinning seizure biology. The development of the structure of the face is influenced by complex molecular interactions between surface ectoderm and underlying developing forebrain and neural crest cells. This influence is likely to continue postnatally, given the evidence of facial growth changes over time in humans until at least 18 years of age. In this case–control study, we hypothesized that people with lateralized focal epilepsies (i.e. unilateral network changes) have an increased degree of facial asymmetry, compared with people with generalized epilepsies or controls without epilepsy. We applied three-dimensional stereophotogrammetry and dense surface models to evaluate facial asymmetry in people with epilepsy, aiming to generate new tools to explore pathophysiological mechanisms in epilepsy. We analysed neuroimaging data to explore the correlation between face and brain asymmetry. We consecutively recruited 859 people with epilepsy attending the epilepsy clinics at a tertiary referral centre. We used dense surface modelling of the full face and signature analyses of three-dimensional facial photographs to analyse facial differences between 378 cases and 205 healthy controls. Neuroimaging around the time of the facial photograph was available for 234 cases. We computed the brain asymmetry index between contralateral regions. Cases with focal symptomatic epilepsy associated with unilateral lesions showed greater facial asymmetry compared to controls (*P *=* *0.0001, two-sample *t*-test). This finding was confirmed by linear regression analysis after controlling for age and gender. We also found a significant correlation between duration of illness and the brain asymmetry index of total average cortical thickness (*r* = −0.19, *P* = 0.0075) but not for total average surface area (*r* = 0.06, *P* = 0.3968). There was no significant correlation between facial asymmetry and asymmetry of regional cortical thickness or surface area. We propose that the greater facial asymmetry in cases with focal epilepsy caused by unilateral abnormality might be explained by early unilateral network disruption, and that this is independent of underlying brain asymmetry. Three-dimensional stereophotogrammetry and dense surface modelling are a novel powerful phenotyping tool in epilepsy that may permit greater understanding of pathophysiology in epilepsy, and generate further insights into the development of cerebral networks underlying epilepsy, and the genetics of facial and neural development.

## Teaser

Balestrini et al. report that people with focal epilepsies caused by unilateral brain lesions, especially of developmental aetiology, have increased facial asymmetry as measured by three-dimensional stereophotogrammetry and dense surface modelling. Facial asymmetry was not correlated with brain asymmetry of regional cortical thickness or surface area.

## Graphical Abstract

**Graphical Abstract:**
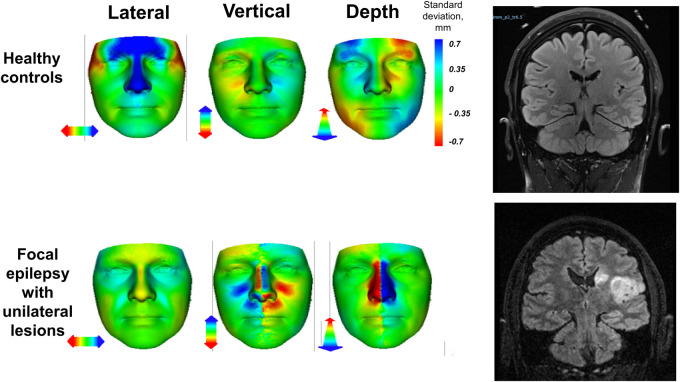

## Introduction

Many genetic syndromes involve facial morphological characteristics, and the facial ‘Gestalt’ can be an important clue in the identification of genetic conditions. Facial morphology reflects environmental influences, as well as individual genomic variation. Atypical face shape can arise from pathogenic genetic variation, as in Down syndrome,^1^ and is an important diagnostic clue in many genetic conditions.^2^ Face and brain development are intricately linked, likely driven by complex molecular interactions between surface ectoderm and underlying forebrain and neural crest cell migration.^3^^,^^4^ Abnormal facial morphology can be associated with underlying brain pathology. At a structural level, the brain exhibits natural asymmetry about the mid-sagittal plane,^5^ exaggeration of which in autism^6^^,^^7^ has its counterpart in quantifiable changes in facial asymmetry.^8^ At a genetic level, genome-wide gene expression is asymmetric from an early developmental stage, and retains differences throughout life.^9^^,^^10^

Facial morphology can also be used to interpret genetic variation: for example, in a set of putatively pathogenic variants, a genetic variant might be considered to have some functional consequence if a particular pattern of quantitative facial change, known to be associated with that variant, is present.^11^ Genome-wide association studies for facial features in Latin Americans have identified several loci, with strong candidate genes, influencing normal facial variation.^12–14^ A novel method to predict face shape variation in people of West African/European mixed ancestry is based on gender, ancestry, and genotype.^15^ Overall, heritability of 60–90% has been estimated for aspects of face shape.^16^

Three-dimensional (3D) stereophotogrammetry has become an important tool in studies of facial shape, dysmorphology, and facial development genetics. It accurately captures the geometry of the face as a mesh of tens of thousands of surface points.^17^ Dense surface models (DSMs) capture variation in facial morphology, based on a dense correspondence of surface mesh points of a set of 3D facial images. Studies using DSM of face shape have delineated facial phenotypes arising from genetic anomaly and teratogen exposure, often enhancing expert dysmorphology review.^18–22^ We previously showed, using DSMs, that individuals with epilepsy and pathogenic structural genetic variants have a significantly more atypical face shape than those without such variants.^23^ The properties of interictal functional networks are substantially different between focal or generalized seizures,^24^ and the timing of network disruption leading to epileptogenesis is largely unknown in both focal and generalized epilepsies. Polygenic contribution has been established for generalized epilepsies, whilst it is less clear for focal ones.^25^^,^^26^ We explored structural asymmetries in the brain and face in the epilepsies, as a step towards further understanding network disruption and pathophysiological mechanisms.

Given the complex molecular interactions between surface ectoderm and underlying developing forebrain and neural crest cells during embryogenesis, likely to continue postnatally,^27^^,^^28^ we hypothesized that, independent from underlying brain asymmetry, people with lateralized focal epilepsies have an increased degree of facial asymmetry, compared with people with idiopathic generalized epilepsy (IGE) or controls without epilepsy, on the basis that lateralized focal epilepsies are currently conceptualized as involving uni-hemispheric networks, whilst IGEs are considered to involve both hemispheres.^29^

## Methods

### Subjects

The study was approved by the relevant ethics committee. Written informed consent was obtained from participants, or informed assent was obtained from parents or guardians. Individuals with epilepsy were consecutively recruited at the National Hospital for Neurology and Neurosurgery (UK). Controls were recruited as volunteers, unaffected relatives of patients or healthy infants, from the UCL Institute of Child Health (London, UK). The data gathered included age, gender, ethnicity, epilepsy diagnosis,^30^ brain MRI results, intellectual disability, and history of facial injury or surgery. Control subjects had no known genetic syndrome, previous facial surgery, or trauma. Non-Europeans were excluded because of the lack of ethnically matched controls. Participants with poor quality surface reconstructions, known bilateral lesions, or unclassified epilepsy, were also excluded. Inclusion and exclusion criteria are summarized in Supplementary Fig. 1.

Epilepsy syndrome classification was based on analysis of seizure semiology, EEG-videotelemetry recording, brain MRI scan, and in some cases, additional investigations such as PET scan and intracranial EEG recording^30^ (Supplementary Data 1).

### Face imaging

For all patients, 3D face images were captured with a single device (Vectra CR 3D; Canfield Scientific Inc.) with the subjects seated, facing directly towards the camera and with the face and chin fully uncovered. A bright target was used to direct gaze, and up to three images were taken with the subject’s face as close to a neutral expression as possible. Two operators (K.C. and S.B.) manually annotated patient images with 22 facial landmarks previously shown to be accurate and reproducible.^31^ The manual annotation was performed blinded to any clinical data. Control subject images were acquired previously with an identical device but annotated by a different operator (P.H.). We assessed intra- and inter-operator reproducibility by randomly selecting 20 images to be landmarked twice by S.B. and K.C. Mean landmark error was <1.5 mm and intra-class correlation coefficients were 0.999–1.000.

A DSM of a set of landmarked face surfaces, described in detail elsewhere,^17^^,^^32^ comprises modes of shape variation arising from a principal component analysis of surface point displacement. Prior to the principal component analysis, using a base mesh (whole face or patch) and aligned sparse landmarks (Supplementary Fig. 2), a dense correspondence of surface points across all faces is computed. The proportion of face shape variance covered by each principal component (PC) is calculated, with PCs ordered in terms of decreasing variance coverage. For a DSM of a collection of faces of varying age, the first PC typically reflects facial growth and correlates strongly with age.^17^ We retained sufficient PCs to cover 99% of shape variance in each constructed DSM (full face and face patches), including both original faces and their mirrored forms (reflection in any plane but usually *x* = 0).

Each face surface captured has as many as 30 000 mesh points. The ‘signature’ of a face surface is the set of position differences at constituent image mesh points from corresponding points on the mean of age/gender-matched healthy controls, normalized against the variation in controls. The ‘signature weight’ of a surface is the square root of the sum of the squared normalized differences across all of the densely corresponded points. Signature weight is a rough estimate of facial dysmorphism. A ‘signature heat map’ visualizes the significance of localized differences using a red–green–blue spectrum with, for example, red and blue reflecting extreme opposite displacements and green coincidence with the mean of the matched controls. Thus, an axis normal to the face surface reflects inward/outward displacement; a lateral (*x*) axis might be used to visualize hypertelorism or dystopia canthorum; a vertical (*y*) axis to reflect unusual nose length; and, a depth (*z*) axis may reflect mid-facial hypoplasia (Supplementary Fig. 3).

The difference between the DSM representations of a face and its mirrored form can also be interpreted as a surface reflecting the ‘raw asymmetry’ of the original face. If a face was perfectly symmetric, the difference would correspond to the mean face in the DSM constituted by faces and their mirrored forms. A signature heat map of an original-mirrored difference can be used to demonstrate the significance of regions of facial asymmetry compared to that of healthy controls. The signature weight of this difference, ‘signature asymmetry index (SAI)’, quantifies degrees of facial asymmetry. Raw asymmetry and signature asymmetry are compared in Supplementary Fig. 4. It is important to note that a raw asymmetry heat map shows differences in millimetres between original and mirrored face surfaces on the original face surface. A signature asymmetry heat map shows differences in standard deviations of the ‘artificial’ surface defined by the original-mirrored difference from similar differences for a set of matched controls.

In order to investigate endophenotypic differences in face shape, we used multi-folded discrimination testing (closest mean; support vector machines; linear discriminant analysis) to determine baseline discrimination rates between controls, focal cryptogenic and focal symptomatic subgroups. After removing individuals with facial injuries, the IGE subgroup size numbered only 27, with almost a 2:1 female:male ratio, which we considered unsuitable for multi-folded discrimination testing. Anthropometric comparisons were made against appropriate age-range matched controls (e.g. noting the narrower age range of the IGE smaller subgroups, with 10 males and 17 females).

### Statistical analysis

For each DSM generated, we calculated means of the differences between original and mirrored facial forms for controls. For both cases and controls, the log of the SAI was normally distributed. All subgroup comparisons of the SAI were tested by two-sample *t*-test and/or ANOVA. A nominal *P*-value <0.05 was considered significant. Bonferroni correction for multiple comparison was applied when appropriate. To identify independent predictors of SAI variation, SAI was considered as the dependent variable in a multivariate linear regression model. This was constructed after adjusting for potential confounding factors, considered as the variables that emerged as significant (*P* < 0.05) in the univariate analyses. Statistical analyses were conducted using SPSS 24 or Stata/IC 11.1.

In a DSM of original and reflected face surfaces, some PCs will represent shape asymmetries.

Those PCs capturing a common asymmetry for a given set of faces will show strong (inverse) correlation between the original faces and their reflections. The PCs can then be ordered for this correlation to determine a league table of those capturing maximal asymmetry for a specific subset of faces. Once an ‘asymmetry’ candidate PC was identified, appropriate left and right-sided anthropometric measures (L and R) were used to compute an associated asymmetry measure (R − L)/(R + L).

### Brain MRI imaging and processing

From the 378 subjects with available facial asymmetry measures, there were T1-weighted MRI scans for 239 subjects taken around the time of acquiring SAI. In total, three different 3D-T1-weighted MRI sequences were used: (i) a coronal T1W 3D inversion-recovery fast spoiled gradient echo (IR-FSPGR) with repetition time/echo time/inversion time = 8.1/3.1/450 ms; field-of-view 187 **×** 240 **×** 240 mm; matrix 170 **×** 256 **×** 256 (176 scans); (ii) an axial T1W 3D (FSPGR BRAVO) with TE/TR/TI 3.6/0.2/400 ms, field-of-view 240 **×** 240 **×** 183 mm, matrix 256 **×** 256 **×** 166, parallel imaging acceleration factor 2 (43 scans); and (iii) a 3D T1-weighted inversion-recovery fast spoiled gradient recalled echo with TE/TR/TI 3.1/7.4/400 ms, field-of-view 224 **×** 256 **×** 256 mm, matrix 224 **×** 256 **×** 256, parallel imaging acceleration factor 2 (20 scans). Sequences 1 and 2 were used for data acquired between August 2004 and March 2013 on a single 3T MRI GE Signa HDx scanner (GE, Milwaukee, WI, USA) using an 8-channel head coil. Sequence 3 was used for data acquired from September 2013 onwards, on a 3T GE Discovery MR750 (GE, Milwaukee, WI, USA) with a 32-channel head coil.

Following a visual inspection, MRI scans with resections were excluded before using FreeSurfer 6.0^33^^,^^34^ for the automated segmentation of brain regions. From the FreeSurfer processed images, we extracted information on 102 brain regions defined in the Desikan-Killiany atlas^35^: 70 regional measures for cortical thickness including average cortical thickness and surface area for each hemisphere, 16 sub-cortical volumes and 26 measures for hippocampal subfields.^36^ Regional measures were adjusted for intracranial volume, age, sex and scanner using linear regression. For an automated quality control step, we employed a script developed by the ENIGMA consortium,^37^ which, for every brain region, identifies potential outliers based on the distribution in the cohort: values showing a z-score of ≥4.7 in either direction were marked as outliers. Following this automated quality control, five highlighted subjects’ segmented brain scans were manually inspected and finally excluded as outliers. Using the resulting 234 scans, we computed the brain asymmetry index (BASI) between contralateral regions, where the BASI of each brain region was defined as the difference between the left and right regional measure, divided by their average. Data on duration of illness was available for 194/234 of these subjects, including focal cryptogenic (*n* = 97), focal symptomatic (*n* = 70) and IGE (*n* = 27).

We computed Pearson’s correlation between duration of illness and the BASI of average thickness and surface area of cortical brain regions to investigate the effect duration of illness has on the brain. We also computed Pearson’s correlation between SAI and the BASI of average thickness and surface area of cortical brain regions.

### Machine learning approach

We aimed to predict subjects’ SAI from the computed 51 BASI brain regions using regularized logistic regression. Specifically, we used the LASSO regularization,^38^ which is a statistical analysis that performs sparse feature selection. BASI values were normalized prior to being used in the learning algorithm: for each feature, the mean in the dataset was subtracted, and the values were divided by the Euclidean norm to ensure that the range for each BASI values was comparable. The dataset was split randomly into 80% training data and 20% test data. We optimized the regularization parameter, alpha, on the training set using 10-fold cross-validation with alpha values ranging from 2^−16^ to 2^−4^. We chose the alpha that led to the best correlation between measured SAI and predicted SAI. Next, we predicted the SAI for samples in the test data using the optimal parameter. This process was repeated 1000 times to account for randomness in splitting train and test data and to better evaluate the trained model’s performance. Model performance for each of the 1000 repetitions was measured using the Spearman’s rank correlation coefficient and the mean squared error (MSE) between measured SAI and predicted SAI. The BrainPainter software was used to visualize the frequency of the brain regions selected across the 1000 repetitions.^39^

In addition to this BASI of brain regions-only model, we sought to improve model performance by incorporating additional features: laterality of the lesion in an MRI scan for symptomatic cases, and the type of epilepsy.

Furthermore, we analysed only focal cryptogenic (*n* = 109) and focal symptomatic (*n* = 96) subjects separately to investigate the prediction accuracy on SAI with LASSO model trained on BASI of brain regions and laterality of the lesion in an MRI scan.

### Data availability

The authors confirm that the data supporting the findings of this study are available from the corresponding author, upon reasonable request.

## Results

### Cohort characteristics

Of the 859 people with epilepsy initially recruited for the study, 378 were included in the analysis and compared with 205 healthy controls. The clinical characteristics of the subjects included in the study are summarized in Table 1.

**Table 1:** Clinical characteristics of all subjects included in the study, including people with epilepsy

Main epilepsy study		
Epilepsy cases, *n* (%)	FS	183 (48)
	FC	145 (38)
	IGE	50 (13)
Mean age in years (range)	Epilepsy cases	40 (18–77)
	Controls	35 (14–73)
Male subjects, *n* (%)	Epilepsy cases	170 (45)
	Controls	86 (42)
Intellectual disability, *n* (%)	Epilepsy cases	101 (27)
History of facial fractures or surgery, *n* (%)		65 (17)

FC, focal cryptogenic unilateral epilepsy; FS, focal symptomatic unilateral epilepsy; IGE, idiopathic generalized epilepsy.

### Discriminating face shape differences between controls and individuals with epilepsy

The faces of the focal cryptogenic and focal symptomatic patient subgroups were strongly distinguishable from controls but much less so from each other, suggesting they have similar degrees (and possibly similar kinds) of facial differences from controls (Table 2). Having established these discriminating differences, consideration of mean face signatures (Supplementary Fig. 5) suggested that outward, horizontal displacement of the inner canthi and vertical displacement of the subnasale were potential discriminating differences. Table 2 summarizes significant reductions found in either or both of palpebral fissure length (suggesting dystopia canthorum) and nose length in focal epilepsy cases but not in generalized epilepsy cases. This was especially so for female (*P* <* *0.001 for palpebral fissure length, *P* <* *0.001 for nose length) rather than male (*P* =* *0.077 for palpebral fissure length, *P* =* *0.099 for nose length) symptomatic cases. Right or left laterality of a symptomatic lesion did not appear to exaggerate these particular face shape differences.

**Table 2:** Multi-folded discrimination analysis to determine baseline discrimination rates between controls and epilepsy subgroups

		Face	Palpebral fissure length		Nose length	
Comparison	Gender (*n*:*N*)	discr	(mm:mm)	*P* (*t*-test)	(mm:mm)	*P* (*t*-test)
CTRL: FC	F (106:45)	0.986	27.8:26.8	0.002**	47.0:46.0	0.108
	M (71:32)	0.943	28.8:28.4	0.292	49.3:47.5	0.049*
CTRL: FS	F (106:59)	0.990	27.8:26.8	2.8E−05**	47.0:44.6	9.4E−04**
	M (71:41)	0.959	28.8:28.2	0.077	49.3:47.9	0.099
CTRL: IGE	F (80:17)		28.0:27.5	0.153	46.6:45.3	0.132
	M (70:10)		28.8:28.5	0.506	49.3:47.7	0.102
FC: FS	F (45:72)	0.732	26.8:26.8	0.935	46.0:44.7	0.107
	M (32:55)	0.697	28.4:28.1	0.442	47.5:47.7	0.871

CTRL, controls; F, female; FC, focal cryptogenic unilateral epilepsy; FS, focal symptomatic unilateral epilepsy; IGE, idiopathic generalized epilepsy; M, male. *P ≤ 0.05; **P ≤ 0.01.

### Signature asymmetry index in individuals with epilepsy

There was no correlation between SAI and age in controls or individuals with epilepsy (Supplementary Fig. 6). There was a significant correlation between SAI and epilepsy duration in all individuals with epilepsy (*n* = 378) (Pearson’s correlation coefficient = 0.245, *P* <* *0.0001), including focal (*n* = 328) and generalized (*n* = 50) types, and this was stronger after excluding individuals with a history of head injury (*n* = 65) (Pearson’s correlation coefficient = 0.312, *P* <* *0.0001). There was no significant correlation between SAI and age at seizure onset. We found significantly higher values of SAI in cases with focal epilepsy (including all unilateral cryptogenic and symptomatic) when compared with controls (*n* = 205) (mean 5.04, SD 0.32 vs 4.95, 0.28, *P* =* *0.0013). This difference was maintained after excluding 53 subjects with a history of facial fractures or surgery (*P* =* *0.0034). There was a significant difference in SAI between cases with focal unilateral symptomatic epilepsy (*n* = 183) compared to controls (mean 5.07, SD 0.30 vs 4.95, 0.28, *P* =* *0.0001). The difference persisted after excluding 27 subjects with a history of facial fractures or surgery (*P* =* *0.0006). We also repeated the comparison of SAI between focal symptomatic cases and controls after removing the 43 cases with clear acquired aetiology (i.e. inflammatory/infectious, tumour, ischaemic/haemorrhage and post-traumatic damage), and we found again a significant difference (mean 5.05, SD 0.29 vs 4.95, 0.28, *P* =* *0.0019). We finally conducted a subanalysis of SAI between focal symptomatic cases with malformation of cortical development (*n* = 45) and controls (*n* = 205), and there was a significant difference (mean 5.10, SD 0.29 vs 4.95, 0.28, *P* =* *0.0015). There was no significant difference in SAI when comparing focal symptomatic cases with hippocampal sclerosis (*n* = 77) and controls (*P* = 0.1687).

**Figure 1:**
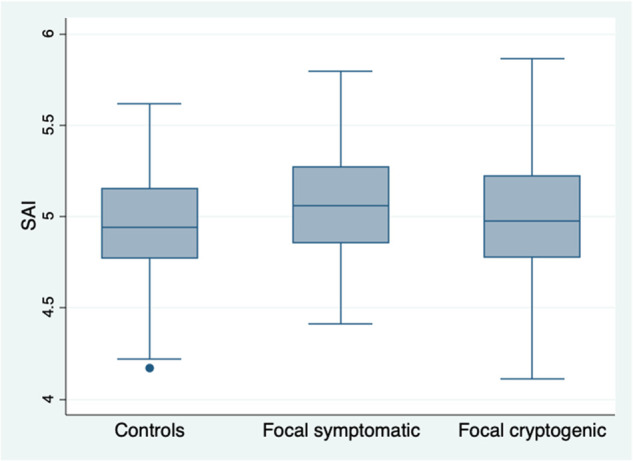
**SAI distribution in focal epilepsies vs controls.** Box plots showing difference in distribution of SAI for cases with focal epilepsy (symptomatic and cryptogenic) and controls: The box includes data from 25th to 75th percentiles, with the median in the middle, the whiskers extend from lower to upper adjacent value and the dots represent outside values.

There was no significant difference in SAI between focal cryptogenic epilepsy cases (*n* = 145) versus controls (mean 4.99, SD 0.33 vs 4.95, 0.28, *P* =* *0.1783); excluding 26 subjects with a history of facial fractures or surgery did not change this observation (Fig. 1). There was no significant difference in SAI between IGE cases versus controls (*P* =* *0.9875), even after excluding 12 subjects with a history of facial fractures or surgery.

**Figure 2:**
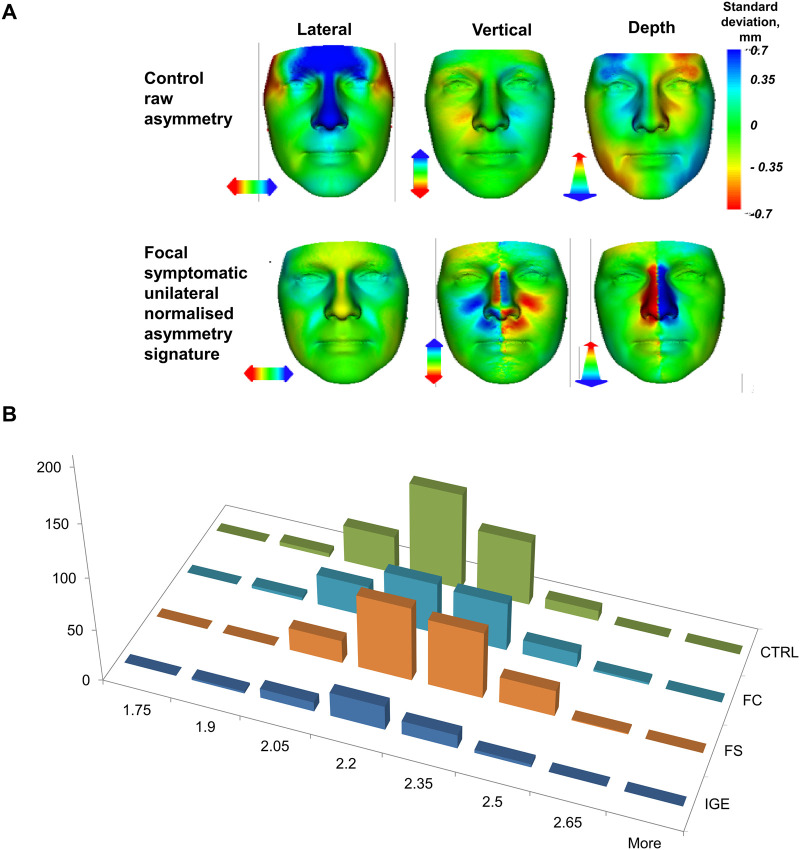
**Raw asymmetry and SAI in controls and epilepsy subgroups.** (**A**) Difference between the average raw asymmetry in the control group (first row) and signature asymmetry in cases with focal symptomatic epilepsy and unilateral lesions (second row). The right dominant depth asymmetry in controls is consistent with the so-called Yakovlevian torque found previously in the brains of typically developing individuals. (**B**) The distribution of SAI in the *x*-axis was compared between each epilepsy subgroup and controls. The most significant facial asymmetry, as measured by SAI, occurs in cases with focal symptomatic unilateral epilepsy. CTRL, controls; FC, focal cryptogenic unilateral epilepsy; FS, focal symptomatic unilateral epilepsy; IGE, idiopathic generalized epilepsy.

One-way ANOVA with Bonferroni correction for multiple comparisons confirmed that the SAI difference between all focal cases and controls was driven by the focal symptomatic unilateral cases (*P* =* *0.001). Multivariate linear regression analysis also confirmed that focal symptomatic unilateral cases were the only category significantly associated with SAI (coefficient 0.089, *P* =* *0.004), after controlling for age (coefficient 0.004, *P* <* *0.001) and gender (coefficient 0.094, *P* <* *0.001) (Table 3, Fig. 2).

**Table 3:** Multivariate linear regression analysis to identify independent predictors of SAI variation

Variable		Coefficient	Standard error	*t*	*P* < |*t*|	95% CI
Age		0.004	0.001	3.62	<0.001**	0.002 to 0.006
Gender (male sex)		0.094	0.025	3.81	<0.001**	0.045 to 0.142
Epilepsy type (controls as reference)	FC	0.029	0.032	0.91	0.362	−0.034 to 0.093
	FS	0.089	0.031	2.88	0.004**	0.029 to 0.151
	IGE	−0.011	0.047	−0.24	0.809	−0.103 to 0.080
Constant		4.882	0.043	114.34	<0.001**	4.798 to 4.966

The model was constructed after adjusting for potential confounding factors, considered as the variables that emerged as significant (*P *<* *0.05) in the univariate analyses.

FC, focal cryptogenic unilateral epilepsy; FS, focal symptomatic unilateral epilepsy; IGE, idiopathic generalized epilepsy. **P ≤ 0.01.

### Specific facial asymmetries in individuals with epilepsy

Table 4 lists face shape PCs and cohort subgroups for which there is a strong inverse (Pearson) correlation (<−0.9) between PC values of corresponding original and reflected faces.

**Table 4:** Correlations (<−0.9) between PC values for original and reflected faces for controls and individuals with epilepsy

PC	10	12	31	55	28	53	61	67	18
CTRL F	−0.997	−0.992	−0.979	−0.971	−0.963	−0.959	−0.934	−0.917	
CTRL M	−0.997	−0.994	−0.984	−0.975	−0.936	−0.952	−0.939	−0.922	
FS F	−0.996	−0.992	−0.976	−0.978	−0.962	−0.959	−0.900	−0.950	
FS M	−0.998	−0.990	−0.975	−0.980	−0.956	−0.961	−0.902		−0.917

CTRL, controls; F, female; FS, focal symptomatic unilateral epilepsy; M, male; PC, principal component.

PC10 involves vertical asymmetry of orbit and ear position as well as lateral displacement of the lower jaw. PC12 captures a horizontal deflection of the nose tip. PC31 depicts a rotation of the nose but about a ‘nasion-subnasale’ axis, with simultaneous vertical displacement asymmetry of the corners of the mouth. These asymmetries are best appreciated as animations (Supplementary Data 2) showing variation between −3 and +3 standard deviations. Other PCs capture asymmetries to a lesser degree and/or reflect less face shape variance (e.g. may not show a strong inverted correlation between original and reflected faces, whilst other PCs are lower down in the PC variance hierarchy, e.g. PC68, PC69 etc.). For the asymmetry delineated in PC12, left-right deflection of the nose, we used the distances between subnasale and left and right exocanthi. Distances between pronasale and left and right cheilion were used for the nose-mouth asymmetry delineated in PC31. Left and right inner canthi distances to nasion were employed to investigate the eye-depth asymmetry as featured in PC67. For the asymmetry mode PC10, we computed the distance between left vs right exocanthi and left vs right mouth corner. As with the palpebral fissure and nose length differences, these specific asymmetries appear to be more significant when the lesion is focal, and in women. Once again, lesion sidedness (right or left-sided lesions) does not appear to exaggerate or bias these asymmetries (Supplementary Table 1).

### Facial and brain asymmetries in individuals with epilepsy

**Figure 3:**
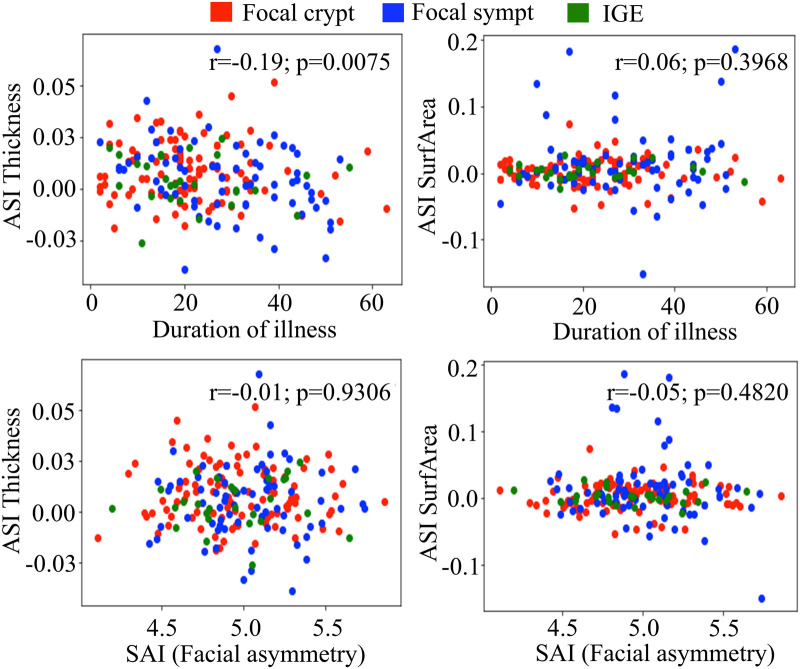
**Correlation analysis of duration of illness and SAI vs BASI average cortical thickness and BASI average surface area.** Duration of illness and BASI average cortical thickness showed *r* = −0.19 (*P* = 0.0075) and for BASI average surface area *r* = 0.06 (*P* = 0.3968). In the case of SAI and BASI cortical measures, the correlation was −0.01 (*P* = 0.9306) and −0.05 (*P* = 0.4820) for BASI average thickness and BASI average surface area, respectively. The strength of relationship shows ASI thickness decreases for longer duration of illness but is different in the case of BASI average surface area and duration of illness.

Duration of illness was significantly correlated with BASI of average cortical thickness (*r* = −0.19; *P* = 0.0075) but not with BASI of surface area (r = 0.06; *P* = 0.39). SAI was neither correlated with BASI of cortical thickness (*r* = −0.01; *P* = 0.93) nor with BASI of surface area (*r* = −0.05; *P* = 0.48) (see Fig. 3).

**Figure 4:**
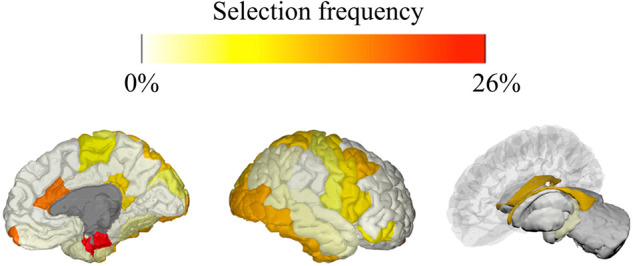
**Brain region selection frequency.** Subjects from all categories were included. The model was trained on BASI brain regions, epilepsy classification and lesion laterality on MRI scan. The entorhinal gyrus (dark red), fimbria (dark orange), pallidum, frontal pole, caudal anterior cingulate (yellow) were the top brain regions selected in the LASSO model to predict SAI across the 1000 iterations.

The LASSO model was trained on subjects of all epilepsy types to predict SAI using the BASI of brain regions and optionally additional features. The main analysis involving all 234 subjects using only the BASI of brain regions yielded an average correlation of −0.022 and an average MSE of 0.099 (Supplementary Fig. 7). Adding the additional non-imaging features yielded a mean correlation of −0.024 and mean MSE of 0.099. Thus, there was no notable difference in the average correlation and MSE of the two models. In addition to making predictions, the LASSO model enabled us to assess which regional BASI were most useful for predicting SAI. Of the 1000 iterations, in 683 models LASSO did not select any features besides the intercept (indicated by correlation values of exactly 0). However, in the remaining models, the most frequently selected features were the BASI of entorhinal gyrus (25.8%), fimbria (25.6%), the pallidum (25.2%), the frontal pole (16.8%), the caudal anterior cingulate (16.5%) and the side of lesion on MRI scan (15%) (Fig. 4; Supplementary Fig. 8). The final analysis was to determine the impact SAI had on focal epilepsies. We analysed only focal cryptogenic and focal symptomatic subjects. Here we found the focal cryptogenic model performed slightly better compared to focal symptomatic cases with average correlation (*n* = 109) of 0.095 (average MSE of 0.11) and average correlation (*N* = 96) of −0.03 (average MSE of 0.09), respectively.

## Discussion

We show that facial asymmetry is greater in individuals with focal symptomatic epilepsy associated with unilateral lesions compared to healthy controls. Actual sidedness of lesion did not appear to increase their impact on the measures. This difference was maintained after excluding individuals with unilateral lesions of established acquired aetiology. Although the size of the lesions was not systematically measured, in most cases the brain structural lesion was not extensive, and its location was deep (e.g. hippocampal sclerosis). Whilst there was a significant effect of disease duration on both brain and facial asymmetries, there was no association between brain asymmetry and facial asymmetry, suggesting that any link between brain disruption and increased facial asymmetry was not due to a direct physical brain asymmetry as measured here. We confirmed our primary hypothesis that people with lateralized focal epilepsies have an increased degree of facial asymmetry, compared with people with IGE or controls without epilepsy, and found that increased facial asymmetry reflects, indirectly, the presence of an underlying focal lesion and the network disruption it might cause.

Our findings of increased degree of facial asymmetry in focal epilepsies associated with unilateral lesions support the perspective that the brain can physically influence the face. In mice, a reduction in brain growth produced an earlier developing and more prognathic face.^40^ Morphometric evidence from both animal models and humans demonstrates that throughout the course of brain growth, correlated variation is produced between the neurocranium and surrounding bony structures, including the face.^41^^,^^42^ In our study, brain and face asymmetries did not correlate. Thus, whilst the disruption to brain physical parameters that a focal symptomatic lesion may cause, a direct physical link seems unlikely to explain our finding that focal epilepsy has greater facial asymmetry when caused by a known underlying unilateral epileptogenic lesion, independent of whether its aetiology is presumptively genetic or acquired. It is possible that the unilateral network disruption accompanying the focal epilepsy leads to the more asymmetric facial development, and that this disruption is more severe in the presence of a brain structural abnormality compared with non-lesional focal epilepsies. There is evidence that remodelling of connectome topology and structurally governed functional dynamics depends on the underlying brain abnormality, for example much more marked networks changes were identified in temporal lobe epilepsy associated with hippocampal sclerosis when compared with temporal lobe epilepsy associated with isolated gliosis, where there were negligible effects on network dynamics.^43^ We found that a developmental aetiology, in particular malformation of cortical development, was significantly associated with facial asymmetry, whilst hippocampal sclerosis of which the aetiology is controversial and most likely multifactorial was not different from controls. This suggests a stronger influence of developmental disorders on facial asymmetry.

Recent work has shown that the brain controls some of the temporal aspects of facial development. The effect of the brain on the timing of facial development has been observed in animal models.^44^ The early influences of the brain on the face may be partially erased during later stages of development^45^; however, some processes may exert different influences at different times. There is evidence of facial growth changes over time in humans until the age of 18 years, including various horizontal and vertical linear measurements and changes in their relative proportions.^27^ Facial dysmorphology may likely arise from aberrant physical interactions between the brain and the face, for example, due to a growth rate that is too rapid, leading to a larger platform for the face to grow on, leaving the face relatively small. Marcucio et al.^46^ hypothesized that the temporal pattern of gene expression within the brain transmits some type of timing information to adjacent tissues.

We found that disease duration is associated with reduced cortical thickness as previously demonstrated,^47^ and with increased facial asymmetry. Widespread progressive cortical thinning exceeding that seen with normal ageing has been found in individuals with focal epilepsy.^48^ Altered connectomic profiling in magnetoencephalography in focal epilepsies has been associated with disease duration.^49^ In our study, people with epilepsy-associated focal brain lesions had greater facial asymmetry compared to controls, paralleling the widespread effect on cortical thickness observed in people with epilepsy and focal brain pathology (i.e. hippocampal sclerosis).^47^ Structural brain asymmetry has been found in other neurological disorders, including autism spectrum disorder where increased facial asymmetry has been described.^8^^,^^50^ We also found correlation between disease duration and facial asymmetry, suggesting an ongoing effect of the epilepsy pathophysiology on facial phenotype, or alternatively that facial abnormalities grow with age.

The primate brain exhibits several directional asymmetries, i.e. Broca’s area in the inferior frontal gyrus is consistently larger and associated with language in the dominant hemisphere.^5^^,^^51^ The most prominent observations of brain hemispheric structural asymmetry include the extension of the right occipital and left frontal lobes across the inter-hemispheric midline and to a shift of structures surrounding the Sylvian fissure, together referred to as the Yakovlevian anticlockwise torque,^52^^,^^53^ and the right frontal and left occipital impressions on the inner surface of the skull, more prominent in right-handers, known as petalia.^52^^,^^54^ Human brain structure is highly determined by an individual’s genotype,^55^^,^^56^ whilst laterality seems influenced by a number of environmental factors in addition to the genetic determinants.^5^ Extreme directional asymmetries associated with brain laterality have been correlated with fluctuating asymmetries in other traits, such as a composite index of ear length and width, ‘atd’ angle, and the widths of wrist, hand, foot and ankle.^57^^,^^58^ Both brain and face asymmetries are likely determined by a complex interaction of environmental and genetic factors.^5^^,^^59^^,^^60^ In the current work, we could not establish the direction of the facial asymmetry, but right- vs left-sided brain lesions did not appear to increase the significance of the facial differences.

For both focal epilepsy subgroups, we found significant discriminating facial differences from controls in terms of reduced palpebral fissure length (possibly dystopia canthorum) and reduced nose length, respectively resulting from outward displacement of the inner canthi and upward displacement of the subnasale. Some focal epilepsies have an established genetic cause, including some focal cryptogenic syndromes [autosomal dominant nocturnal frontal lobe epilepsy^61^; genetic epilepsy with febrile seizures plus^62^^,^^63^; autosomal dominant partial epilepsy with auditory features^64^; familial focal epilepsy with variable foci^65^] and some focal symptomatic syndromes associated with malformation of cortical development such as focal cortical dysplasia.^66–71^ A recent whole-exome sequencing study found a significant enrichment of ultra-rare deleterious variants in established epilepsy genes in individuals with familial non-acquired focal epilepsy, including cryptogenic and hippocampal sclerosis cases.^72^ Morphogenetic events during brain and face development are controlled and coordinated by extracellular signalling pathways, such as Sonic Hedgehog (SHH), bone morphogenetic protein, fibroblast growth factor, Nodal, and retinoid signalling.^73^ Signalling from the ventral midline is critical for normal midface development, a process in which SHH plays a key role.^74^ Although the exact molecular mechanisms that regulate *SHH* expression are not known, various genes are implicated in normal and abnormal variation of face and brain.^4^ We speculate that the same genetic changes can affect both brain and facial development.

We did not find increased facial asymmetries in people with IGE. This epilepsy type, including the electroclinical syndromes of childhood absence epilepsy, juvenile absence epilepsy, and juvenile myoclonic epilepsy, has a substantial genetic component.^25^ However, the exact genetic architecture underlying these common epilepsies remains to be elucidated. A recent case–control exome sequencing study revealed a significant excess of ultra-rare deleterious variation in known epileptic encephalopathy genes in patients with familial genetic generalized epilepsy and non-acquired focal epilepsy^72^ showing how genetic risk may arise from ultra-rare variants of large effect, including *de novo* mutations, in a small proportion of common generalized epilepsies, as in the rare epilepsies.^75^ It remains unclear why (mono) genetically determined common IGEs are less severe than epileptic encephalopathies and why we did not find significant facial alteration in this epilepsy type. We speculate that there is a less asymmetric underlying genetic effect, and less asymmetric network disruption in IGE, such that there is no observed increase in facial asymmetry. Also, the current conceptualization of IGE includes the presence of bilateral epileptogenic networks compared to unilateral networks involved in focal epilepsies; however, asymmetric focal volumetric brain changes have been found in IGE.^47^

There is a series of limitations in our study. The first is the categorization of epilepsy syndromes. Epilepsy classification evolves continuously, and the current findings might also help in this direction. We chose the old 1989 classification^30^ based on our initial hypothesis that the cause of focal epilepsy may differently affect the degree of facial asymmetry. The latest ILAE classification^76^ states that structural conditions should be considered not directly ‘genetic’ but despite there being a genetic basis, the structural correlate underpins the person’s epilepsy (for example in focal symptomatic cases with presumably genetic lesions including malformations of cortical development or dysembryoplastic neuroepithelial tumour^66^^,^^77^). Autism spectrum disorder is a relatively frequent co-morbidity in people with epilepsy and is known to affect facial asymmetry^8^; however, we could not include this factor in the analysis as not all the patients in our cohort had an adequate assessment to confirm or exclude a diagnosis of autism. Recent studies did not find a significant genetic correlation at the common variant level between autism and epilepsy in a large epilepsy cohort and, on this basis, we may speculate that facial asymmetry may be led by different mechanisms in the two conditions.^25^^,^^78^ We found some gender differences in facial asymmetry in individuals with epilepsy but not in controls; this is difficult to interpret given the relatively small sample size and the unclear evidence in the literature.^79^ There was a high number of missing data for disease duration, hence we could not control for this in relation to facial asymmetry; however, this was a cross-sectional study with limited value in interpreting facial measures longitudinally. Other limitations include limited data availability for controls (i.e. lack of handedness data) and unmeasured size of brain lesions.

We propose that the greater facial asymmetry in cases with focal epilepsy caused by unilateral abnormality might be explained by early unilateral network disruption, and that this is independent of underlying brain asymmetry. These findings suggest that DSM models of facial asymmetry could represent a powerful novel phenotyping process in epilepsy that will permit greater understanding of pathophysiology in epilepsy, and generate further insight into the mechanisms of network disruption during facial and neural development.

## Supplementary material

Supplementary material is available at *Brain Communications* online.

## Funding

S.B. was supported by the Muir Maxwell Trust and the Epilepsy Society. S.M.L. was supported by an Engineering and Physical Sciences Research Council Doctoral Training Partnership studentship (EP/R513143/1). A.A. holds a Medical Research Council eMedLab Medical Bioinformatics Career Development Fellowship. This work was partly supported by the Medical Research Council [MR/L016311/1]. UCB provided financial support for R.D. UCB had no editorial control and no input or decision over the selection of authors or topics discussed.

## Competing interests

The authors have no competing interests to declare.

## Supplementary Material

fcab068_Supplementary_Data

## Abbreviations

BASI =: brain asymmetry index
DSM =: dense surface modelling
IGE =: idiopathic generalized epilepsy
MSE =: mean squared error
PC: = principal component
SAI =: signature asymmetry index
