# Supplementary material for: Increased facial asymmetry in focal epilepsies associated with unilateral lesions
Source: Brain Commun. 2021 Apr 19;3(2):fcab068. doi: 10.1093/braincomms/fcab068 (PMC8244637; doi:10.1093/braincomms/fcab068)
Supplement: fcab068_Supplementary_Data [file fcab068_Supplementary_Data.zip › Supplementary_material.docx]

**Increased facial asymmetry in focal symptomatic epilepsy associated with unilateral lesions**

**Supplementary Data**

**Supplementary Data 1 - Epilepsy syndromes and genetic conditions**

The epilepsy syndrome classification was defined according to the Commission on Classification and Terminology of the International League Against Epilepsy (1989):

*focal cryptogenic* (*FC*) if an epileptogenic focus was localised but the cause was not demonstrable on brain MRI scan of at least 1.5T;

*focal symptomatic* (*FS*) if an underlying structural lesion was shown to be the cause of epilepsy through at least EEG-videotelemetry recording, if not intracranial EEG;

*idiopathic generalised epilepsy* if all seizures were generalised (absences, myoclonic jerks and generalised tonic-clonic

*(IGE)* seizures), with generalised bilateral, synchronous, symmetrical, ictal and interictal EEG abnormalities; not associated with structural brain lesions on MRI or abnormal neurological symptoms and/or signs interictally, with normal neuropsychological status (assessed by neuropsychometric evaluation);

*unclassified* none of the above or unclear syndromic classification (i.e. focal epilepsy with lesion on brain MRI scan not associated with the epilepsy focus; generalised epilepsy with abnormal neuroimaging and neurological examination, and/or developmental delay; multifocal epilepsy).

The focal symptomatic cases included the following conditions: hippocampal sclerosis (n=77), malformation of cortical development (n=45), ischaemic/haemorrhage (n=16), dysembryoplastic neuroepithelial tumour (n=13), tumour (n=12), inflammatory/infectious (n=6), cortical lesion of unclear aetiology (n=4), arteriovenous malformation (n=3), cystic lesions (n=3), post-traumatic damage (n=2), hamartoma (n=1), Sturge-Weber syndrome (n=1).

**Supplementary Table 1.** **Comparison of four asymmetry parameters selected in relation to the PC mode values indicating facial asymmetry: subnasale deflection (PC12); nose-mouth asymmetry (PC31); mouth swivel (PC10); and, inner-canthal depth (PC67).**

|  |  |  |  |  |  | |  | | |  | | |  | | | | |  | |  |
| --- | --- | --- | --- | --- | --- | --- | --- | --- | --- | --- | --- | --- | --- | --- | --- | --- | --- | --- | --- | --- |
| Comparison | Sex (n : n) |  | Subnasale | |  | |  | | Nose-Mouth | | | | | | |  | | |  |  |
|  |  |  | Deflection | | *p* (t-test) | |  | | | | Asymmetry | | | | *p* (t-test) | | | |  | |
| CTRL F: M | - (106:71) |  | 0.0047 | 0.0017 | 0.214 |  | | 0.0078 | | | | 0.0073 | | 0.835 | | |  |  |  |  |
| CTRL : FC | F (106:45) |  | 0.0047 | 0.0032 | 0.569 |  | | 0.0078 | | | | 3.0E-05 | | **0.004** | | |  |  |  |  |
|  | M (71:32) |  | 0.0017 | 0.0004 | 0.691 |  | | 0.0073 | | | | -0.0018 | | **0.021** | | |  |  |  |  |
| CTRL : FS | F (106:72) |  | 0.0047 | -0.0006 | **0.019** |  | | 0.0078 | | | | 0.0004 | | **0.001** | | |  |  |  |  |
|  | M (71:55) |  | 0.0017 | 0.0006 | 0.710 |  | | 0.0073 | | | | -0.0022 | | **0.002** | | |  |  |  |  |
| CTRL : Unil FS | F (106:59) |  | 0.0047 | -0.0012 | **0.015** |  | | 0.0078 | | | | 0.0009 | | **0.004** | | |  |  |  |  |
|  | M (71:41) |  | 0.0017 | 0.0031 | 0.643 |  | | 0.0073 | | | | -0.0028 | | **0.002** | | |  |  |  |  |
| CTRL : IGE | F (80:17) |  | 0.0015 | 0.0040 | 0.480 |  | | 0.0070 | | | | 0.0037 | | 0.318 | | |  |  |  |  |
|  | M (70:10) |  | 0.0020 | 0.0065 | 0.459 |  | | 0.0077 | | | | 0.0104 | | 0.635 | | |  |  |  |  |
| Unil FS L : R | F (32:27) |  | -0.0029 | 0.0007 | 0.338 |  | | 0.0007 | | | | 0.0011 | | 0.906 | | |  |  |  |  |
|  | M (20:21) |  | 0.0016 | 0.0045 | 0.542 |  | | 0.0049 | | | | -0.0008 | | 0.430 | | |  |  |  |  |

| Comparison | Sex (n : n) |  | Mouth | | |  | |  | | Inner-Canthal | |  |  |  |
| --- | --- | --- | --- | --- | --- | --- | --- | --- | --- | --- | --- | --- | --- | --- |
|  |  |  | Swivel | | | *p* (t-test) | |  | | Depth | | *p* (t-test) | |  |
| CTRL F: M | - (106:71) |  | 0.0033 | 0.0034 | 0.925 | |  | | 0.0124 | | 0.0093 | 0.443 | | |
| CTRL : FC | F (106:45) |  | 0.0033 | -0.0011 | **0.010** | |  | | 0.0124 | | -0.0032 | **2.7E-04** | | |
|  | M (71:32) |  | 0.0034 | -0.0004 | **0.050** | |  | | 0.0093 | | -0.0023 | **0.019** | | |
| CTRL : FS | F (106:72) |  | 0.0033 | -0.0001 | **0.033** | |  | | 0.0124 | | -0.0052 | **4.0E-07** | | |
|  | M (71:55) |  | 0.0034 | 0.0001 | 0.097 | |  | | 0.0093 | | -0.0056 | **2.3E-04** | | |
| CTRL : Unil FS | F (106:59) |  | 0.0033 | -0.0008 | **0.013** | |  | | 0.0124 | | -0.0040 | **8.0E-06** | | |
|  | M (71:41) |  | 0.0034 | 0.0011 | 0.281 | |  | | 0.0093 | | -0.0053 | **0.002** | | |
| CTRL : IGE | F (80:17) |  | 0.0033 | 0.0064 | 0.326 | |  | | 0.0117 | | 0.0014 | 0.063 | | |
|  | M (70:10) |  | 0.0036 | 0.0022 | 0.675 | |  | | 0.0100 | | 0.0149 | 0.414 | | |
| Unil FS L : R | F (32:27) |  | -0.0006 | -0.0011 | 0.831 | |  | | -0.0050 | | -0.0029 | 0.681 | | |
|  | M (20:21) |  | -0.0020 | 0.0041 | 0.055 | |  | | -0.0043 | | -0.0063 | 0.764 | | |

**Supplementary Figure 1. Diagram showing study design, with inclusion and exclusion criteria.**

**Supplementary Figure 2.** Example of annotation with 22 aligned sparse landmarks (adapted from Chinthapalli et al, 2012). Landmarks A–F are in the midline. Landmarks 1–8 are paired and only shown for the right side of the face. They are as follows: A = nasion; B = pronasale; C = subnasale; D = labiale superius; E = labiale inferius; F = gnathion; 1 = exocanthion; 2 = palpebrale superius; 3 = endocanthion; 4 = palpebrale inferius; 5 = ala nasi; 6 = christa philtri; 7 = cheilion; 8 = lower auricular attachment. Landmark 8 is applied to all unprocessed face surface images, but the ears are omitted from the base mesh because of variable loss of surface at the image periphery because of occluding hair.

**
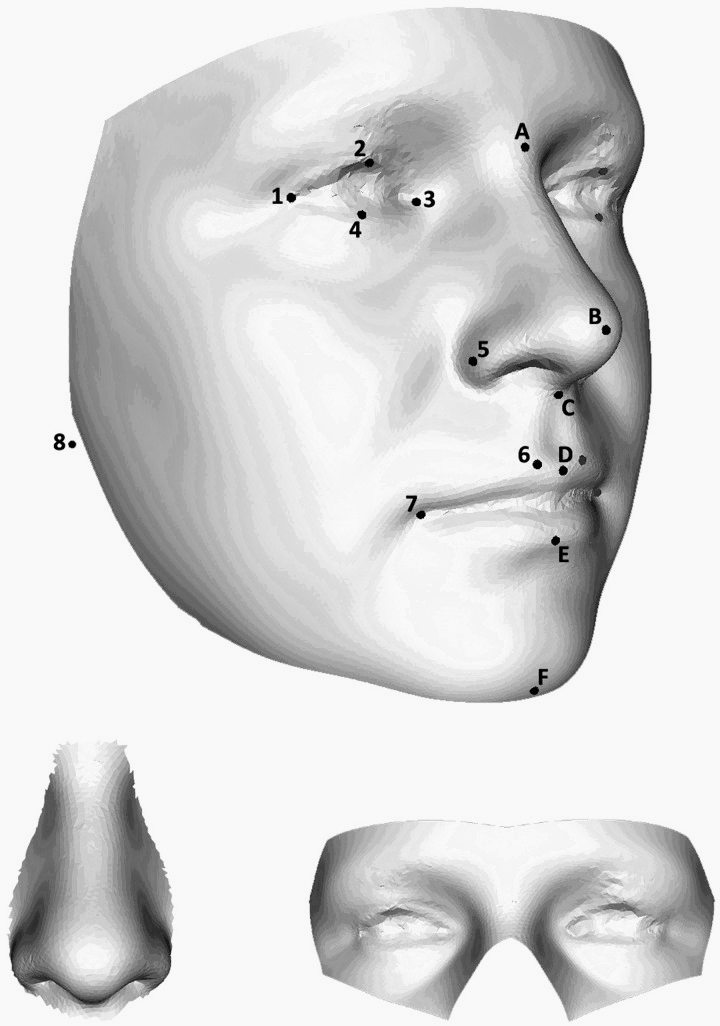
**

**Supplementary Figure 3.** Heat map for the same face for each of the three axes, illustrating x-axis/lateral displacement of eyes (slight hypotelorism with eyes different colours), y-axis-vertical displacement for long face (forehead blue and chin red), and z-axis/depth displacement with prominence of nasal bridge (blue) and flattened zygomatic arch (red cheek bones).


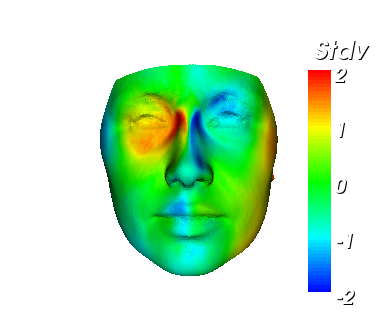

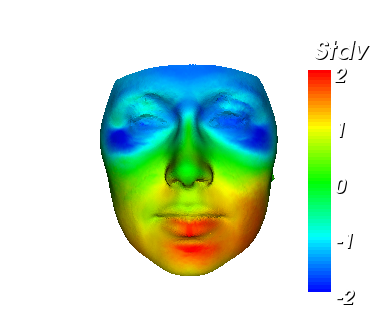

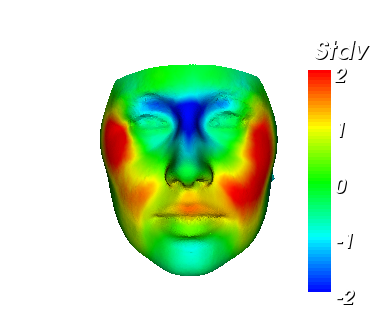

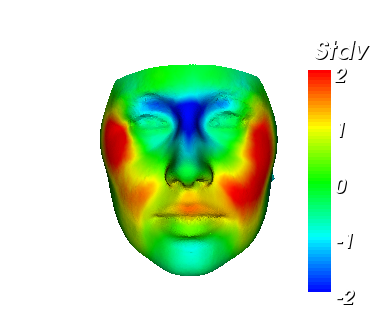

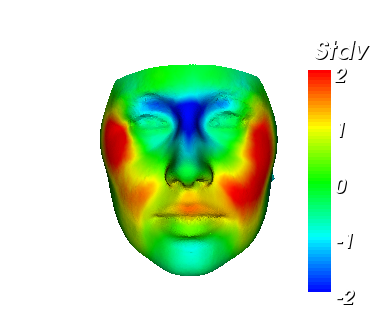

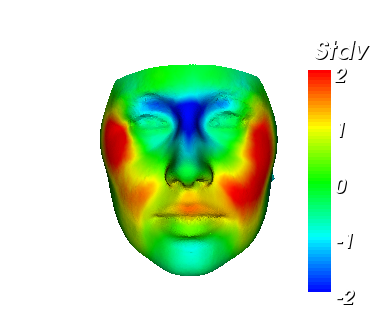


**Supplementary Figure 4. Comparison between raw asymmetry and signature asymmetry. A and B show raw asymmetry heat maps (measured with the same scale, in millimetres) along lateral, vertical and depth axes for the mean control face (row 1) and for a randomly selected case (row 2). C shows normalised signature asymmetry (in SDs) for the same case in B, for the same axes. A shows that the mean control has some lateral displacement of the nose and supraorbit to the left side of the face (col 1), a much smaller upward left displacement (col 2) and a right dominant forward displacement (col 3). The lateral and depth displacements are concordant with the previously identified Yakovlevian torque of the control brain (Herbert et al., 2004) and asymmetry of the control face (Hammond et al., 2008). C shows a raw right displacement of the chin (col 1). Given the control mean has no chin displacement, C (signature asymmetry) shows this as very significant. Note the middle (i.e. upward) difference in B (col 2) is a very slight upward displacement on the right side which in C (col 2) is shown as quite significantly different from the mean control, which has a left-sided upward displacement. The depth difference for this example is similar to the control mean except on the tip of the nose.**

**Supplementary Figure 5.** Heat mapped facial signatures for average male and female faces (by column) of control and patient subgroups (by row). Green regions of the face indicate close correspondence with controls; red and blue extremes correspond to difference from controls at 1 and -1 standard deviations respectively.


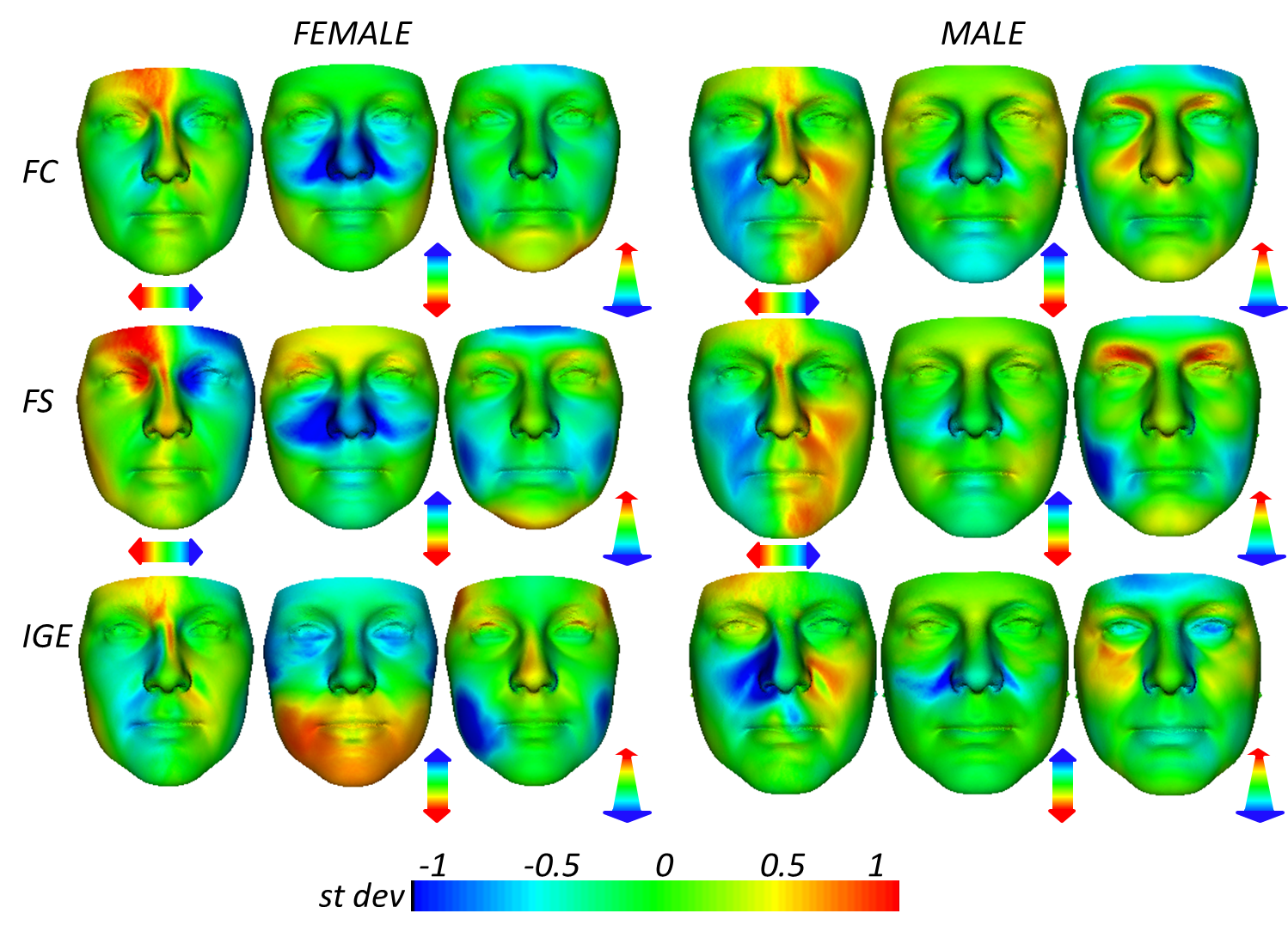


**Supplementary Figure 6.** Scatter plots of SAI vs age for each epilepsy type compared with controls (t-test significance).


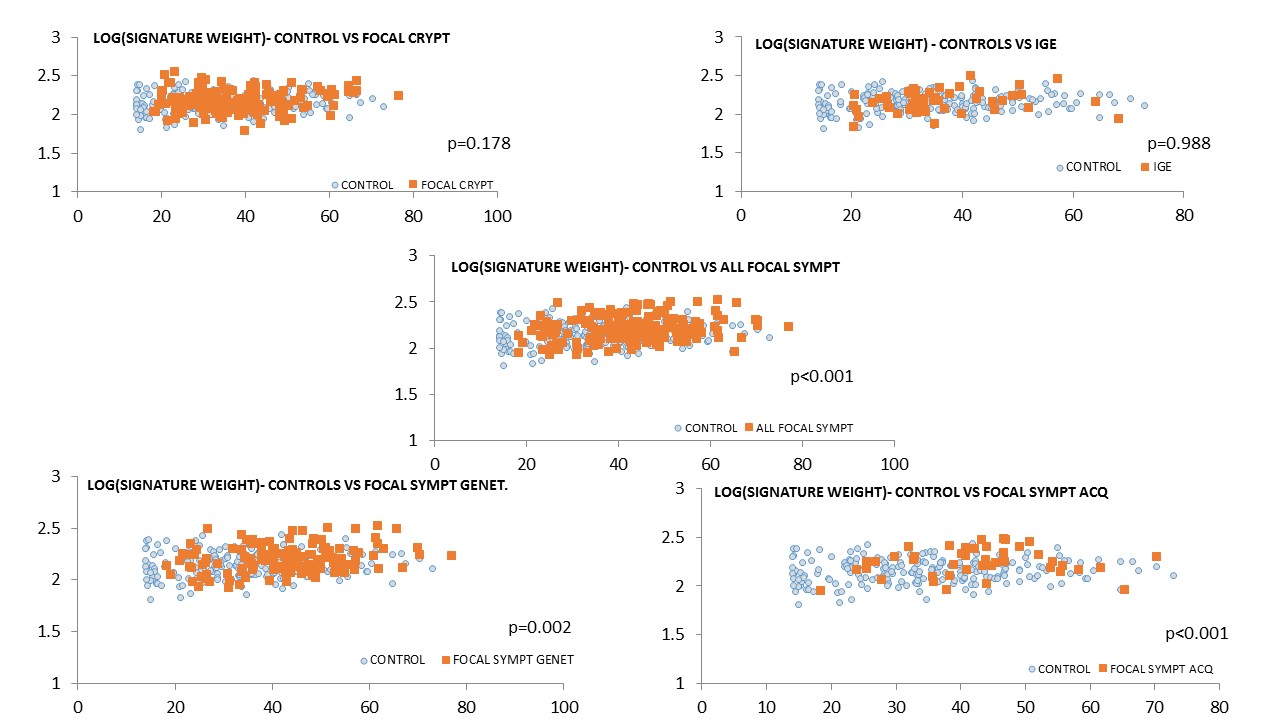


**Supplemental Figure 7. Correlation and MSE distribution of predicted vs actual SAI. Average correlation of predicted vs actual SAI with model trained on BASI of brain regions, epilepsy category and lesion laterality on MRI scan were -0.02 and average MSE was 0.097. This indicates a potential ambiguous relationship between BASI of brain regions atrophy and SAI observed in people with epilepsy.**


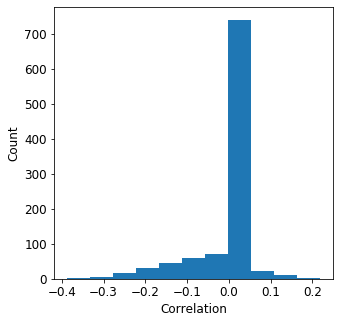

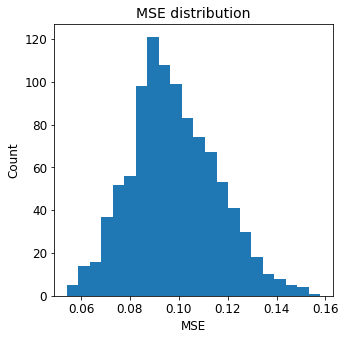


**Supplemental Figure 8. Features selection frequency: Subjects from all categories included, model trained on BASI of brain regions, epilepsy category and lesion laterality on MRI scan. The entorhinal gyrus (258), fimbria (256), pallidum (252), frontal pole (168), caudal anterior cingulate (165), and side of lesion on MRI (150) were the top features selected in the LASSO model to predict SAI across the 1000 iterations. These features are part of the hippocampus sub-fields, cortical and subcortical regions of the brain and even side of lesion present indicating some link of lateral or contra-lateral impact on SAI.**


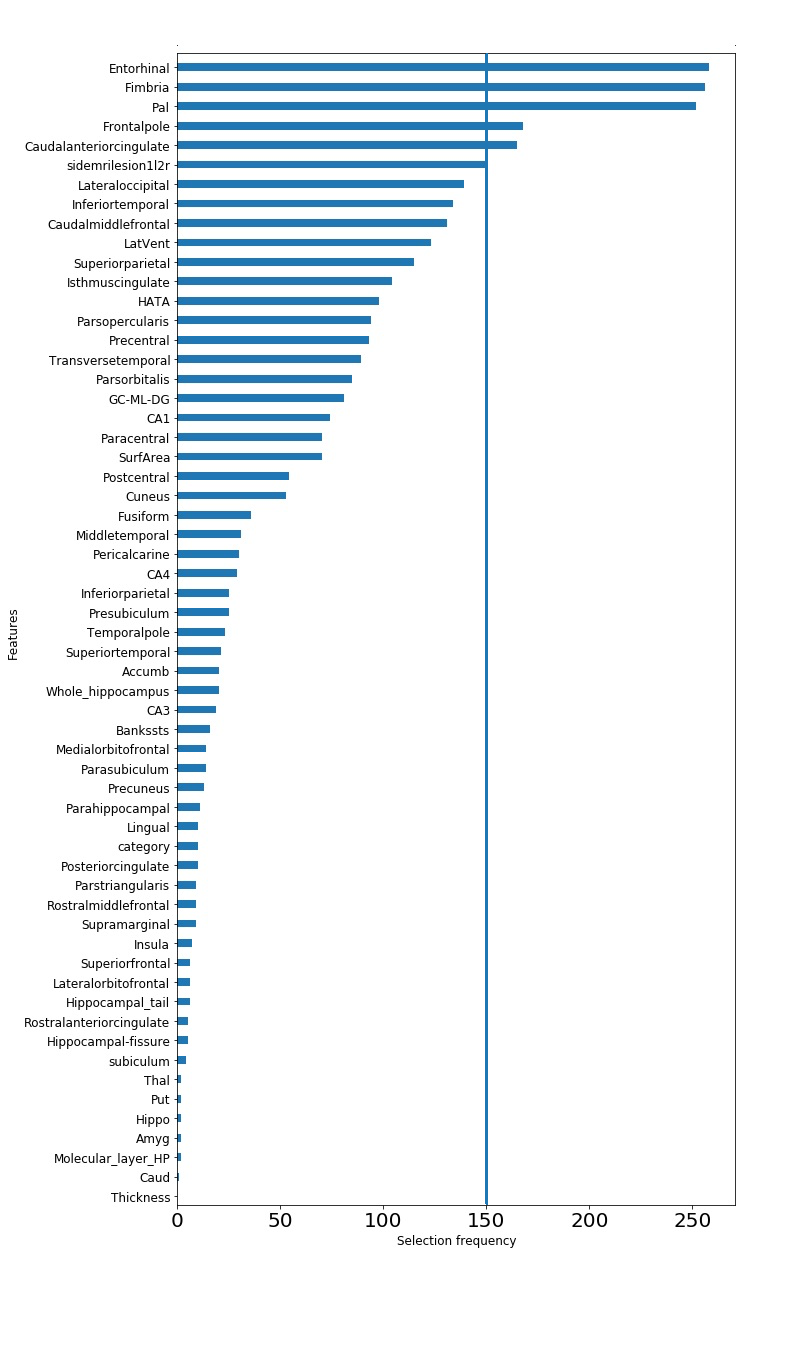


**References**

Chinthapalli K, Bartolini E, Novy J, et al. Atypical face shape and genomic structural variants in epilepsy. Brain. 2012;135(10):3101-3114.

Commission on Classification and Terminology of the International League Against Epilepsy. Proposal for Revised Classification of Epilepsies and Epileptic Syndromes. Epilepsia 1989; 30: 389–399. doi:10.1111/j.1528-1157.1989.tb05316.
